# How Property Markets Determine Welfare Outcomes: An Equilibrium Sorting Model Analysis of Local Environmental Interventions

**DOI:** 10.1007/s10640-016-0101-8

**Published:** 2017-02-13

**Authors:** Amy Binner, Brett Day

**Affiliations:** School of Social Sciences and International Studies, University of Exeter, Exeter, EX4 4RJ UK

**Keywords:** Equilibrium sorting models, Property market, Welfare analysis, Tenure choice, Distribution

## Abstract

This paper examines the pivotal role played by property markets in determining the magnitude and distribution of welfare changes resulting from localised environmental change. We address that issue using an equilibrium sorting model (ESM) calibrated, by way of example, to the circumstances of a road infrastructure project in the English town of Polegate. Previous ESM research has tended to assume that all households rent property from a fixed property stock. The narrative that arises from those models concerns environmental gentrification, wherein access to environmentally improved locations is appropriated by the relatively wealthy through their ability to out-compete the less wealthy in the rental property market. Our research shows that to be only part of a much more complex story. We develop a model that extends the sophistication with which ESMs replicate property market dynamics, allowing for households to choose whether to rent or purchase their home and introducing greater realism into housing supply responses to changing market conditions. Our research shows that property markets redistribute welfare gains across the population in complex ways in which tenure choice and housing supply constraints play central roles.

**Electronic supplementary material:**

The online version of this article (doi:10.1007/s10640-016-0101-8) contains supplementary material, which is available to authorized users.

## Introduction

The environmental impacts of many projects and policies are highly localised. Consider as examples, the creation of a wind farm, the siting of an incinerator plant, the closure of a landfill site or, the project that motivates this paper, the construction of a bypass that directs road traffic around rather than through a town. Until recently, typical appraisal practice has been to assume that the merits of such projects could be measured by estimating the welfare changes experienced by current residents of an impacted location. Over recent years, researchers have expressed increasing concern that this assumption may be a poor reflection of the true welfare outcomes (Goulder et al. 2003; Smith and Carbone 2007; Carbone and Smith 2008; Bayer et al. 2009; Klaiber and Phaneuf 2010). At the root of those concerns is the fact that the standard approach provides only a partial equilibrium analysis that ignores potentially important behavioural responses to changing environmental quality. The purpose of this paper is to explore perhaps the most significant of such responses, those that take place through decisions made in the property market.

Following a tradition of research that dates back to Tiebout (1956), economists have made significant progress in exploring behavioural responses mediated by property market decisions (Sieg et al. 2004; Kerry Smith et al. 2004; Tra 2010; Klaiber and Phaneuf 2010). The key tool in advancing that research effort has been the development of *equilibrium sorting models* (ESMs) (Epple and Platt 1998; Bayer et al. 2004; Ferreyra 2007; Kuminoff et al. 2010). The basic structure of an ESM is to envisage a property market where households choose a residential location from a finite set of neighbourhoods that differ with regards to their level of provision of public goods (of which environmental quality is one element). Equilibrium in the market is established through adjustments in property prices that match supply with demand in each neighbourhood. Since ESMs are based on an explicit model of household preferences, they provide a framework within which to consider the welfare impacts of interventions that result in localised changes in public good provision (Smith et al. 2004; Sieg et al. 2004; Ferreyra 2007; Walsh 2007; Klaiber and Phaneuf 2010; Tra 2010). Insomuch as ESMs explicitly allow for behavioural adjustments to those policy changes through decisions in the property market, ESMs provide *general equilibrium* (GE) welfare measures to contrast with the *partial equilibrium* (PE) measures used currently for project appraisal (Sieg et al. 2004; Smith et al. 2004; Walsh 2007).

Over recent years the field of equilibrium sorting modelling has advanced significantly along various fronts (Kuminoff et al. 2010), although certain characterisations of the property market adopted by many ESMs remain highly simplistic. For example, it is standard practice to assume that all households are renters and that the rents they pay leave the economy under study, accruing to absentee landlords. Accordingly, ESMs tend to ignore the fact that many households own their own homes and that some may also receive rental income as landlords from their ownership of rental property. Moreover, the manner in which ESMs handle housing supply involves considerable abstraction; houses are treated as bundles of building-block-like homogenous quality units that, in response to property market changes, can be costlessly repackaged to create new configurations of properties. The virtues of this abstraction have been questioned previously in the literature (Epple and Platt 1998; Bayer et al. 2004). We review these assumptions in detail in Sect. 2 of the paper.

Where ESMs have been used to explore the welfare outcomes of localised environmental change, the reliance on these simplifying assumptions supports a particular story of property market responses. For example, Banzhaf and Walsh (2004) and Sieg et al. (2004) show that improvements in the environmental quality of a neighbourhood leads to *environmental gentrification*; households currently renting properties in that neighbourhood find themselves displaced by wealthier households more able to afford the increased rents. Consequently, an environmental project that seems to offer hope of welfare gains to the less wealthy residents of the improved area may actually condemn them to welfare losses since rising rents may force them to take up residence in another, less-preferred neighbourhood. Careful consideration reveals that that narrative rests quite heavily on the simplified representation of the property market. For instance, in the real world many households own rather than rent their homes. Indeed, for homeowners in an improved neighbourhood increasing property prices are anything but a burden. As prices rise and their properties grow in value, such homeowners realise capital gains which open up new welfare-enhancing consumption opportunities either within or outside the property market.

The central contribution of this paper is to present an ESM that allows us to relax a number of the standard simplifying characterisations of the property market that have been used in previous analyses and so examine the consequences of those assumptions for welfare analysis. In Sect. 3, we describe that model in detail. Drawing on the work of Binner and Day (2015), we describe an ESM with tenure decisions allowing households the option of renting or purchasing when choosing where to live. In addition we develop an alternative representation of the housing supply function that better captures the realities of accommodating changing populations in spatially limited neighbourhoods. In Sects. 4 and 5 we explore the implications of these modelling advances by means of a simulation analysis in which the model is calibrated to reflect the features of a real intervention; the construction of a bypass around the rural town of Polegate in England.

The simulations reported in this paper provide a number of important insights. First, they confirm that the magnitude and distribution of welfare effects predicted by a PE analysis grossly misrepresent those identified by the GE analyses enabled by an ESM. What is clear is that policy decisions that naively attempt to direct local public goods provision for the purpose of achieving redistributive aims (as has been advocated in the environmental justice literature; for example, Walker and Bickerstaff 2000; Liu 2000; Walker 1998; Walton and Shaw 2003; Poustie 2014) may have unanticipated and potentially counter-productive outcomes. Second they show that while environmental gentrification is part of the story of these GE responses to local environmental changes, it is by no means the whole story. It is not only a household’s income, but also their tenure status that determines the welfare changes they experience as property markets adapt to localised environmental changes. They also demonstrate that welfare outcomes are greatly influenced by the nature of the housing supply response to the changing conditions resulting from a localised environmental change. Indeed, while previous research has tended to focus on demand-side responses (Binner and Day 2015; Tra 2010; Ferreyra 2007; Smith et al. 2004; Sieg et al. 2004), our work shows that supply-side responses play a significant role in shaping the predictions of welfare change arising from ESM analyses.

## Property Market Representations in Equilibrium Sorting Models

Early ESMs sought to shed light on the processes leading to the formation of cities (Mills 1967), the migration of individuals across communities (Tiebout 1956; Oates 1969; Ellickson 1971) and the segregation of populations across space (Schelling 1969). Over recent years, the complexity of ESMs and the range of applications in which they have been applied has expanded rapidly (Kuminoff et al. 2010). In particular, ESMs have been employed in the evaluation of policies relating to air quality (Smith et al. 2004; Sieg et al. 2004; Tra 2010), private school vouchers (Ferreyra 2007), urban sprawl prevention (Walsh 2007) and property tax reforms (Binner and Day 2015). Meanwhile, recent methodological papers have extended the literature by seeking to address moving costs (Kuminoff 2009; Bayer et al. 2009; Epple et al. 2010; Ferreira 2010), dual market decisions (Kuminoff 2011) and dynamic optimisation (Fernandez and Rogerson 1998; Epple and Ferreyra 2008; Epple et al. 2010).

Despite very considerable advances, numerous fundamental aspects of choice in property markets remain unexplored. One rather significant but widely-maintained assumption adopted by the vast majority of ESM analyses is that all households are renters (Fernandez and Rogerson 1998; Sieg et al. 2004; Klaiber and Phaneuf 2010). Epple and Platt (1998) explore an ESM with renters and owners, but within their model tenure status is exogenously determined and the tenure distinction is incidental to the paper’s central focus on income sorting across neighbourhoods. Of course, tenure status is not exogenous but rather the subject of active choice. Indeed, empirically it is well-documented that that self-selection process leads to marked differences in the characteristics of households in different tenure groups; for example, owners have been observed to be wealthier (Dietz 2003; Dietz and Haurin 2003), have improved health (Rohe and Leslie 1996; Rohe et al. 2001) and have higher-achieving children (Green and White 1997; Haurin et al. 2002).

There are good reasons to suspect that tenure has a very important role to play in understanding the welfare implications of local changes in environmental quality. Imagine, for example, a localised improvement in the environment that increases the desirability of a neighbourhood, putting upward pressure on local property prices and rents. For homeowners the outcome is unequivocally good. Not only do they get to enjoy an improved neighbourhood but they also benefit from the appreciating value of their home. Realising their capital gains by selling their property might enable them to achieve greater welfare by moving to a previously unaffordable larger property in some other neighbourhood. Renters, on the other hand, find that the benefit they enjoy from an improved environment is immediately offset by increases in their rents. What is more, since self-selection leads to income-sorting in tenure, these differential impacts on renters and owners may well have important distributional implications.

A second area worthy of consideration concerns the way in which ESMs handle housing supply. The usual practice is to assume that housing can be defined as a homogeneous good that can be purchased at a constant per unit price within a neighbourhood (Epple and Romer 1991; Epple and Platt 1998; Bayer et al. 2004; Epple et al. 2001; Ferreyra 2007). In effect, households “assemble” a property in their preferred location by buying-up units of the housing good. The quantity of housing units purchased by a household can be thought of as approximating real life choices over the size and quality of housing. At a given price, if a household reduces their consumption of housing units then their total expenditure on housing falls. Likewise, households can increase the number of housing units they consume but will have to spend more on housing. One justification for this approach is based on Sieg et al. (2002) who demonstrated that when housing enters the utility function through a sub-function that is homogeneous degree one, it is possible to construct a “housing quantity” index tantamount to an empirical analogue to the homogeneous housing unit.

Within ESMs the standard approach is to consider a housing supply function in which the number of units available within a neighbourhood is non-declining in the per unit housing price. Of course, the nature of the supply response depends critically on the time horizon under consideration. In the short term housing supply is likely to be highly inelastic; a fact which motivates the conventional ESM assumption that the quantity of housing units remains fixed in the event of changing market conditions (Epple et al. 2010; Fernandez and Rogerson 1998; Epple and Platt 1998). However, that same conventional approach assumes that individual housing units can be traded-off, transferred between households, and reconfigured to create new properties of different sizes and qualities. This means that while the overall aggregate quality of the housing stock is unchanged, individual households are able to increase or decrease the quality of the housing that they consume. In this regard, the standard approach assumes that we can costlessly move for example from a neighbourhood of *N* households each with a property offering *X* / *N* units of the housing index to a neighbourhood of $N+1$ households each enjoying a property offering $X/\left( {N+1} \right)$ units of the housing index. In other words, new households can be accommodated within the current housing stock without cost. We refer to this as costless repackaging. In reality, housing supply adjustments are achieved through a combination of home improvements, depreciation, house-sharing, remodelling and relocation. The housing supply function must capture the confluence of these activities in the property market. The precise effect that this would have on the total number of quality units is unclear but it is not obvious that dividing a property of N units into two would result in two properties of N/2 units.

In this paper we address these shortcomings through an ESM that we describe in the next section. That ESM introduces a choice over renting and owning, allows for households to earn income from rental property in the economy and explores an alternative specification of the housing supply function that better captures medium to long term supply responses. In particular, it allows for (i) elastic supply such that more housing units can be constructed but with increasing marginal costs (as per, Epple et al. 2010; Fernandez and Rogerson 1998; Hallstrom and Smith 2003; ii) a capacity constraint for the total quantity of development that can occur within a neighbourhood and (iii) a cost associated with repackaging housing units. Capturing those realities in the housing supply function makes a substantive difference to the patterns of market adjustment predicted by an ESM. Our paper challenges ESM analysts to consider more deeply the implications of the standard, mathematically-convenient supply-side assumptions.

## The Model

The ESM used in this research is a development of that first described in Binner and Day (2015), where a model which endogenised tenure choice was used to explore the impacts of reforms of US taxation policies concerning mortgage interest payments. In this section, we outline the key elements of that model and the extensions developed for this research, though we delay explicit description of those extensions until we come to examine their impacts in the simulation exercise described in Sect. 5.

### The Economy

We imagine a set of households indexed $i=1, \ldots ,I$ living in a geographic region divided into a set of spatially discrete neighbourhoods, $j=1, \ldots , J$. Each neighbourhood differs in its residential desirability according to differences in the levels of *K* local public goods, $\left\{ {g_{j,1} , g_{j,2} ,\ldots ,g_{j,K} } \right\}$. Households are also heterogeneous and differ in their incomes, *y*, preferences for quantity of housing, $\beta$, and preferences for homeownership, $\theta$. Preferences for homeownership are motivated by a number of factors including ideology, social status, perceived stability, and expectations over future house prices.^1^ The distribution of household types across the population is defined by the joint multivariate density function, $f\left( {y, \beta , \theta } \right)$. The economic problem we explore concerns demand and supply decisions in this economy’s property market.

### The Demand Side

Households’ property market decisions have a number of dimensions. First, the discrete choices of location and tenure where the *location choice* is between the *J* neighbourhoods (migration outside of these neighbourhoods is not considered) and the *tenure choice* between the options of renting, *R*, and owning, *O*. Defining the set of tenure options as $T= \left\{ {R, O} \right\}$ with elements indexed *t*, a household’s residential choice is identified by a neighbourhood and tenure bundle, $\left\{ {j, t} \right\}$. Second, households choose how much to spend on housing. Following previous treatments (Epple and Romer 1991; Epple and Platt 1998; Bayer et al. 2004; Epple et al. 2001; Ferreyra 2007), housing is defined as a homogeneous good that can be purchased at a constant per unit price within a neighbourhood, $p_j$. As discussed in Sect. 2, households “assemble” a property in their preferred location by buying-up units of the housing good, *h*; a characterisation intended to approximate real-world choices over the size and quality of housing. The number of units demanded by household *i* when choosing to live in neighbourhood *j* under tenure type *t* is denoted,

1 $$\begin{aligned} h_{i,j,t}= & {} h\left( {p_j , g_j ;y_i , \beta _i ,\theta _{i,t} } \right) \nonumber \\ \forall i= & {} 1,\ldots ,I,\quad j=1,\ldots ,J, \quad t=O,R \end{aligned}$$

where $$p_j $$ and $$g_j $$ are respectively the unit property price and index of local public goods (to be defined shortly) for neighbourhood *j*. As a result of the discrete nature of the choice, when a household makes the residential choice $$\left\{ {j,t} \right\} $$, we observe $$ h_{i,k,s} =0, \forall \left\{ {k,s} \right\} \ne \left\{ {j,t} \right\} $$.

To become a homeowner, a household must take out a mortgage on which mortgage interest, $$m_i$$, is due. Differences in the mortgage rate across households can be interpreted as representing differing abilities of households to secure a mortgage and bargain for cheaper interest rates. Mortgage interest is paid only on the amount borrowed, which is equal to the product of the loan-to-value ratio, $$\delta _i$$, and the value of the housing purchased, $$p_j h_{i,j,t} $$. Differences in the loan-to-value ratio are driven by differences in incomes and are included to reflect households’ differing abilities to fund property purchases from their personal wealth.^2^ Total demand for housing amongst households choosing $$\left\{ {j,t} \right\} $$ is calculated by integrating over all households,

2 $$\begin{aligned} H_{j,t}^D =\int \int \int h\left( {y, \beta ,\theta _t } \right) f\left( {y,\beta ,\theta _t } \right) dyd\beta d\theta _t \end{aligned}$$

In a standard ESM households are all renters. Once we introduce tenure we must also allow for the possibility of capital gains. Capital gains (or losses) occur when an owner sells some or all of their housing at a price higher (lower) than the amount that it cost them to purchase it. A further discussion of capital gains is provided in Sect. 5.2.

### The Supply Side

Our ESM examines the ramifications of a policy that results in a localised change in public good provision. We assume that prior to the change the property market has achieved a long-term equilibrium under the initial conditions. In that equilibrium, each neighbourhood *j* is home to $$n_j^0 $$ households who between them demand $$H_j^0 $$ units of housing, where $$ H_j^0 $$ is the sum of housing demand from households choosing to rent and households choosing to own.

To model the property market adjustments resulting from changing market conditions, assumptions must be made regarding the supply response of housing. A frequent assumption in the ESM literature is that housing supply is perfectly inelastic (Epple et al. 2010; Fernandez and Rogerson 1998; Epple and Platt 1998) such that the number of housing units remains fixed at $$H_j^0 \left( {j=1, \ldots , J} \right) $$. While that may be an accurate description of the short term, our interest is in medium to long-term responses in which it is reasonable to assume that housing supply responds to changes in the neighbourhood property price, $$p_j $$. Indeed, an alternative assumption to be found in the ESM literature is a housing supply curve that passes through $$H_j^0 $$ and is as increasing function of $$p_j $$ (Epple et al. 2010; Fernandez and Rogerson 1998; Hallstrom and Smith 2003). Of course, that specification allows for the possibility of unlimited construction of new housing units within a neighbourhood at increasing property prices, this supply function does not capture the reality of spatially-constrained urban development. In addition, the standard assumption in ESM models is that as the population of a neighbourhood $$\left( {n_j } \right) $$ changes, the housing units within that neighbourhood can be costlessly repackaged to create the properties demanded by the new residents. In the UK, towns and cities are space constrained and empirically the elasticity of housing supply has been shown to be low (Swank et al. 2002; Meen 2005). This affects the number of new properties that can be constructed as well as the number of households that can be accommodated within an area in the short to medium term.

Accordingly, our specification of the housing supply function contains two additional elements. First a development capacity, $$D_j $$, that places an upper limit on the quantity of housing units $$(h_j )$$ that can be constructed in a neighbourhood (that is, $$h_j \le D_j )$$. Second, we include an element which captures the costs of repackaging housing units. That element is an increasing function of the change in the number of residents in a neighbourhood, $$ {\Delta }n_j =\left| {n_j - n_j^0 } \right| $$, and hence an indicator of the degree of repackaging required to create the properties needed by those new residents. The housing supply function in general form is given by;

3 $$\begin{aligned} H_j^s =h^{s}\left( {p_j , H_j^0 ,D_j , {\Delta }n_j } \right) \quad \forall j=1,\ldots ,J \end{aligned}$$

### Rental and Purchase Prices

In the model described here, we assume that the market maintains a single price for housing units, whether those units are being sold for rent or purchase. Such an outcome would arise as a result of arbitrage activities if buyers and sellers experience identical costs in both ownership and rental markets, and provided the owners or constructors of housing units face no restrictions as to the sector into which they can supply their property. The decision to model rental and purchase prices as being identical within a neighbourhood was driven by a number of factors including (i) that properties in the UK can transition from being rental to purchase with relative ease such that suppliers could increase their profits by altering the tenure of their property if prices were not equal, (ii) that mortgage interest and loan to value ratios already drive a wedge between rental and purchase costs, and (iii) our calibration of preferences for homeownership implies that not every household prefers to be a homeowner. In our model, we assume that the price of housing units is inclusive of maintenance costs, which would be included in the actual price charged to renters or anticipated as an unavoidable cost by buyers.^3^

A standard assumption in ESM applications is that the stock of rental properties are owned by a landlord (or landlords) outside the economy to whom all rents accrue. An alternative assumption would be to allow households in the economy to be owners of some or all of the rental stock. When households are also landlords, changes in market conditions that lead to changes in rental prices will have implications for household income providing another pathway through which welfare can be redistributed through the property market. While we experimented with models that allowed for rental stock ownership, the functional form assumptions adopted in this paper (to be discussed shortly) preclude income effects of this type. Accordingly in this paper we maintain the standard assumption of absentee landlords.

### Government

In our model, the government’s role is to implement projects that impact on the provision of local public goods.^4^ We also consider the impact of government interventions that seek to compensate for damages resulting from such projects. The particular policy we seek to replicate is that based on the UK’s Land Compensation Act 1973, whereby homeowners are entitled to compensation for reductions in the value of their housing that result from environmental damage generated by public works.

### Local Public Goods

Households derive utility from the combined provision of local public goods, which is represented by an index of local public goods provision,

4 $$\begin{aligned} g_j = \mathop \sum \limits _{k=1}^K \gamma _k g_{j,k} \end{aligned}$$

such that$$ \gamma _k $$ is the weight placed on the $$k{\mathrm{th}}$$ element in $$g_j $$. For simplicity, in the calibrated simulation exercise developed in the following sections, we assume that each neighbourhood provides just two exogenous public goods, $$g_{j,1} $$ and $$g_{j,2} $$, resulting in the following public goods index,

5 $$\begin{aligned} g_j =g_{j,1} +\gamma g_{j,2} \end{aligned}$$

where $$\gamma $$ is the weight that households place on $$g_{j,2} $$ relative to $$g_{j,1} $$. It is assumed that $$\gamma $$ is uniform across all households and between neighbourhoods, such that households agree on the ranking of neighbourhoods in terms of the desirability of their local public good provision.

### Household Optimisation

Households derive utility from local public goods, $g_j$, housing, *h*, and other consumption, *c*. Tenure status affects the utility enjoyed by a household from the flow of services provided by housing. All else equal, household *i* derives the same level of utility from owning *h* units of housing as from renting $\theta \left( h \right)$ units. For simplicity and clarity, households are assumed to have the same preference for local public goods, $\alpha$. Household utility is defined by the function,

6 $$\begin{aligned} U_{i,j,t}= & {} U\left( {h,c;y_i ,\alpha ,\beta _i , \theta _{i,t} , g_j } \right) \nonumber \\ \forall i= & {} 1,\ldots ,I, \quad j=1,\ldots ,J, \quad t=O,R \end{aligned}$$

The optimisation problem of household *i* can be decomposed into two stages. First, households calculate their optimal housing and consumption choices for each neighbourhood and tenure bundle. The conditional maximisation problem is,

7 $$\begin{aligned}&\mathop {\max }\limits _{\left( {h,c\hbox {|}j,t} \right) } U\left( {h,c;y_i ,\alpha ,\beta _i , \theta _{i,t} , g_j } \right) \nonumber \\&\quad s.t. y_i =\left\{ {{\begin{array}{ll} p_j h_{i,j,t} +c_i &{} \quad t=R \\ \left( {1+m_i \delta _i } \right) p_j h_{i,j,t} +c_i &{}\quad t=O \\ \end{array} } } \right. \end{aligned}$$

The solutions to (7) yield the set of conditional indirect utility functions,

8 $$\begin{aligned} V_{i,j,t}= & {} \left\{ {{\begin{array}{ll} {V\left( {p_j , g_j ;y_i , \alpha , \beta _i , \theta _{i,R} } \right) }&{} \quad { t=R} \\ {V\left( {p_j , g_j ;y_i , \alpha , \beta _i ,\theta _{i,O} ,m_i , \delta _i } \right) }&{} \quad {t=O} \\ \end{array} }} \right. \nonumber \\ \forall j= & {} 1,\ldots ,J,\quad t=O,R \end{aligned}$$

In the second stage, a household’s optimal residential choice is identified as the neighbourhood, tenure bundle, $\left\{ {j,t} \right\}$, that provides the maximum value for (8).

### Equilibrium

An equilibrium of the model is defined by a mapping of each household to one neighbourhood and an associated vector of property prices, $p=\left\{ {p_1 ,\ldots ,p_J } \right\}$, such that;

- Each household resides in the neighbourhood that maximises its utility given the equilibrium vector of prices, *p*, and public good provision, *g*.
- All housing markets clear,
  $$\begin{aligned} H_{j,t}^S =H_{j,t}^D \quad \forall j=1,\quad \ldots ,J,\quad t=O, R \end{aligned}$$

The underlying properties that support the existence of equilibria in ESMs are unaltered by the introduction of an endogenous tenure choice and preferences for homeownership. Namely, the single crossing, boundary indifference, ordered bundles and stratification assumptions are sufficient to ensure the existence of equilibria (Epple et al. 2010; Epple and Platt 1998).

## Model Calibration

To explore the model, we developed a computer realisation of the ESM using the Matlab programming environment^5^ making particular assumptions regarding functional forms and parameter values. In essence, the model is used to simulate the decisions of a set of households predicting which neighbourhood and tenure option they will choose under particular market conditions.

The model described in this paper was developed as part of a research project carried out for the UK’s Department for Transport (DfT). The particular assumptions made in calibrating the model were designed to replicate a real DfT project; construction of the A27 bypass around the town of Polegate in southern England. The project sought to achieve a high-degree of realism by defining a multi-neighbourhood economy closely resembling Polegate’s actual urban organisation (Binner 2012). To provide greater clarity in understanding the key mechanisms that drive the complex array of adjustments that occur in the property market following a localised change in environmental quality, the research recorded in this paper reports on a simplified, two-neighbourhood version of the analysis. Accordingly, while our simulations are rooted in reality, they are only a loose approximation to that reality.

In the following, we summarise the assumptions made in calibrating the two-neighbourhood model. The calibration draws heavily on the DfT’s Post Opening Project Evaluation (POPE) A27 Polegate Bypass report (2009) which describes how the noise environment in different neighbourhoods changed following construction of the bypass in 2002. To calibrate the model to the situation prior to the bypass, hereafter the *baseline*, we draw on census data from 2001 and data on property sales in Polegate over the period 1995–2012 to estimate a hedonic property price model. As described below, the parameters of that hedonic were important in calibrating the ESM. The hedonic price model was estimated using a spatial and temporal smoothing estimator to account for changes in the shape of the function over time, endogenous sorting and omitted spatial covariates.

### The Economy

For the purposes of the simulation, the economy is divided into two regions; the town centre and the suburbs. The town centre is closer to a range of amenities (which might include parks, shops, schools etc.), however, they are also exposed to greater road noise as a result of traffic that, in the first instance, passes through the town centre on the old main road (B2247). The suburbs and town centre were defined as groupings of census tracts and, according to 2001 census data for those tracts, both neighbourhoods had approximately equal populations and homeownership rates of around eighty per cent. As shown in Table 1, we take those as the conditions characterising the baseline property market equilibrium prior to the building of the bypass.

**Table 1:** Baseline characteristics of the neighbourhoods

	Town centre	Suburbs
Population share	0.5	0.5
Homeownership rate	0.77	0.79
Average distance from centre (m)	400	1100
Road noise level (dB)	59	40
Price per housing unit (£)	5183	5258
Public goods index	36.9	38.0
Housing units per property	Approx. 2.0	Approx. 4.1

### Property Prices

Following the method discussed in Sieg et al. (2002), baseline neighbourhood prices for a unit of homogeneous housing were derived from the Polegate hedonic property price model, estimated using a temporal and spatial smoothing estimator, as the neighbourhood-specific intercepts for the year 2000. Those unit prices were higher in the relatively quieter suburbs, £5258 per unit, than in the town centre, £5183 per unit.

### Households

Household utility is represented by a Cobb Douglas utility function,

9 $$\begin{aligned} U_{i,j,t} = g_j^\alpha \theta _{i,t} (h_{i,j,t} -\omega _t )^{\beta _i }c_i ^{1-\alpha -\beta _i } \end{aligned}$$

Household preferences for public goods, $\alpha$, and homeownership, $\theta _{i,t} =\left\{ { \omega _t , \theta _{i,t} } \right\} \forall i, t$, are assumed to be independent of their income and housing expenditure. Notice that with a Cobb Douglas utility function $, \beta _i$ can be interpreted as the share of income that household *i* commits to purchasing housing. As such, expenditure on property remains constant provided income does not change.

We drew 1600 households, replicating the number of households affected by the bypass (Highways Agency 2009), from a joint bivariate distribution, $f\left( {\ln \left( y \right) , \beta } \right)$, of logged incomes, $\hbox {ln}\left( y \right)$, and preferences for housing, $\beta$. The parameters of the joint distribution were estimated using the breakdown of gross weekly income and expenditures on housing from the Expenditure and Food Survey 2001–2002. The resulting parameters were,

10 $$\begin{aligned} \left( {\mu _{\ln \left( y \right) } ,\mu _\beta } \right)= & {} \left( {9.83, 0.17} \right) \nonumber \\ {\Sigma }_{\ln \left( y \right) ,\beta }= & {} \left( {{\begin{array}{ll} {2.72}&{} \quad {-0.07} \\ {-0.07}&{} \quad {0.03} \\ \end{array} }} \right) \end{aligned}$$

where $\mu _{\ln \left( y \right) }$ and $\mu _\beta$ are the means of $\hbox {ln}\left( y \right)$ and $\beta$ respectively and ${\Sigma }_{\ln \left( y \right) ,\beta }$ is the corresponding variance–covariance matrix.

To calibrate household preferences for public goods, $\alpha$, we use the technique detailed by Carbone and Smith (2008). To implement that procedure we require estimates of willingness to pay (WTP) for improved access to the town centre and WTP for reduced noise pollution. We approximate the former using the implicit price of proximity to the town centre from the Polegate hedonic model and the latter using the implicit price for noise pollution estimated in Day et al. (2007). The calibrated value of $\alpha$ is 0.11.

In the absence of detailed information on loan-to-value (LTV) ratio by income group, the LTV ratio was calibrated using data from the FSA Mortgage Product Sales Data Trends Report (2007) which summarizes the proportion of homeowners in each LTV bracket. LTV ratios (ordered from lowest to highest) were assigned to households (ordered from highest income to lowest) and a mean zero random component was added to the loan-to-value ratio. Preferences for homeownership, $\theta _{i,t}$ are normalised against renting such that,

$$\begin{aligned} \omega _t =\left\{ { {\begin{array}{ll} 0&{} \quad {\hbox {if}} \ \ t=R \\ {0.02}&{} \quad {\hbox {if}} \ \ t=O \\ \end{array} }} \right. \end{aligned}$$

and

$$\begin{aligned} \theta _{i,t} =\left\{ { {\begin{array}{ll} 1&{} \quad {\hbox {if}} \ \ t=R \\ {\theta _i }&{} \quad {\hbox {if}} \ \ t=O \\ \end{array} }} \right. \end{aligned}$$

where $\theta _i$ is drawn from a log normal distribution;

11 $$\begin{aligned} \hbox {ln} \left( {\theta -0.71} \right)\sim & {} N\left( {\mu _\theta , \sigma _\theta ^2 } \right) \nonumber \\ \left( {\mu _\theta ,\sigma _\theta ^2 } \right)= & {} \left( {0, 0.06} \right) \end{aligned}$$

Both $\omega _t$ and the mean and variance parameters, $\mu _\theta$and $\sigma _\theta ^2$, were calibrated using a maximum likelihood procedure that, as described subsequently, also established values for other unknown parameters.

### Local Public Goods

Neighbourhoods are differentiated by their distance to the town centre and road noise levels. The local public goods index for neighbourhood $j \left( {j=1, 2} \right)$ is given by,

12 $$\begin{aligned} g_j =g_{j,1} +\gamma g_{j,2} +\xi _j \end{aligned}$$

where $$g_{j,1} $$ is (negative) distance from Polegate town centre and $$g_{j,2} $$ is noise level in decibels, dB. The proximity measure was calculated using ArcGIS as the average road travel distance from properties in a neighbourhood to the town centre. Road noise was measured using the average 18-h decibel level. Noise levels before and after the bypass in the two neighbourhoods were calibrated using information from the A27 Polegate Environmental Statement (1991). Following the same process as was used to calibrate the parameter $$ \alpha $$ (Carbone and Smith 2008) we derive a value for $$\gamma $$ of 0.02.

The public goods index also contains an unobserved element, $\xi _{j } \left( {\textit{for}\, j=1, 2} \right)$, which serves to capture public goods that have been omitted from our simplified specification. Those unobserved elements of the public good were calibrated in the same maximum likelihood procedure used to establish the parameters of the distribution of preferences for homeownership. Given those values, the public goods indices calculated from (12) suggest that the predicted level of public good provision is greater in the suburbs, consistent with the higher property prices observed in that neighbourhood.

### The Baseline and the Localised Environmental Change

The calibration exercise allows us to establish conditions in the property market in Polegate before the bypass was built. Those conditions are summarised in Table 1. As we have discussed, where possible, details of the two neighbourhoods including populations, homeownership rates, housing unit prices, road noise and proximity to amenities are taken directly from data. Likewise details of the population including the distribution of income, loan-to-value ratios and ownership of rental property are derived from empirical sources. Given our assumptions regarding the structure of preferences empirical estimates of preferences for housing, $\beta _i$, preferences for road noise, $\gamma$, and preferences for public goods $\alpha$ can also be estimated from data. As already indicated, the final step in calibrating the model is to establish values for unknown parameters. In our model, those concern preferences for homeownership, $\theta _{i,t}$ and $\omega _t$, and unobserved quality characteristics of neighbourhoods, $\xi _j$. We establish those through a maximum likelihood procedure that, given a particular set up to our model, seeks values for the unknown parameters that match the model’s predictions of equilibrium conditions most closely with the observed data in the baseline. Finally, the number of housing units demanded by each household, to form the property they choose in the baseline, is calculated by solving for housing demand at the baseline prices using the Marshallian demand function.

Notice from Table 1 that road noise levels are higher in the town centre (59 dB) than in the suburbs (40 dB). Properties in the town centre neighbourhood are, on average 400 m from the centre of the town, whereas properties in the suburbs are an average 1100 m from the centre. Using our derived value for $\gamma$ of 0.02, the retrospective values of the public goods indices are 36.9 for the town and 38.0 for the suburb.

The Polegate bypass constructed in 2002, directs through-traffic away from the city centre along a bypass that skirts the town suburbs. The noise exposure of properties in the town centre fell by 2 dB to 57 dB as a result of the bypass. In the suburbs road noise rose by 1 dB to 41 dB (Highways Agency 2009). According to our calibrated model, those changes were sufficient to reverse the ordering of the public goods index making the town centre a more desirable residential location than the suburbs in terms of public goods provision.

## Property Market Simulations

We use the calibrated model to explore the role of the property market and, more particularly, modelling assumptions regarding the functioning of that property market on the distributional outcomes of localised environmental change. To do that, we present the results of a series of simulations each of which is progressively more sophisticated in the way it represents the property market and its response to changing conditions. In each case we begin by calibrating the particular property market representation to the data in Table 1 to establish property market conditions and household residential choices in the baseline. Subsequently, the model simulates households’ location and tenure choices under the changed conditions resulting from the bypass construction, using iterative techniques to identify a new set of prices that achieve equilibrium in the property market (Lagarias et al. 1998).^6^ We begin by introducing the standard property market characterisation in Sect. 5.1 and introduce endogenous tenure choice in Sect. 5.2. In Sects. 5.3 and 5.4 we address housing supply constraints and include fixed transaction costs associated with moving house, hereafter moving costs. A range of alternative moving costs were explored, however, for the sake of brevity and because that feature of property markets has been studied elsewhere (Kuminoff 2009; Bayer et al. 2009; Epple et al. 2010; Ferreira 2010), we confine further details to the online supplementary information in Appendix B.

### Standard Property Market Characterisation (SESM)

We begin by examining a simulation that adopts the standard property market characterisation used in the ESM literature. In this model, all households are renters and the number of housing units in each neighbourhood is fixed but, in response to changing conditions, those units can be costlessly repackaged to create new properties of different sizes. The demographics of the two neighbourhoods derived from calibrating this model to baseline conditions are presented in Table 2.

**Table 2:** Standard ESM—calibrated baseline neighbourhood composition

	Town centre	Suburbs
Price	5183	5258
Population	798	802
Population share	0.5	0.5
*Population characteristics*		
Mean income	57,528	84,647
Mean $\beta$	0.36	0.10

A number of sorting patterns are evident. First, households with higher incomes tend to locate in the suburbs. Attracted by the higher provision of public goods, these households compete up the price of properties in this neighbourhood. Second, poorer households, who spend a larger proportion of their income on housing, are attracted to the town centre by its relatively low property prices. Likewise, those preferring properties with more housing units, as captured by the preference parameter $\beta _i$, tend to locate in the town centre where property is cheaper.

The conventional, partial equilibrium (PE) approach to project appraisal identifies the costs and benefits of the changes in the noise environment as the welfare impacts immediately experienced by the households under their baseline residential choices.^7^ The top panel of Table 3 summarises welfare measures from the PE analysis, reporting the average welfare change experienced by renters in the two neighbourhoods and the total welfare change for the bypass project. Benefitted by the fall in road traffic noise, residents of the city centre enjoy substantial welfare gains. The opposite is true for those in the suburbs who lose welfare as a result of their greater exposure to traffic noise. Since the partial analysis does not allow for property market adjustments either in prices or residential location choices, the aggregate welfare impacts it predicts is simply the benefits realised by the town centre residents net the losses experienced by residents of the suburbs; an overall welfare gain of £81,461. For a policy maker interested in the distributional consequences of the scheme the story is pretty straightforward. According to the PE analysis welfare gains flow exclusively to households in the town centre. Since those households are generally poorer than households in the suburbs, the bypass construction project appears progressive in nature.

The general equilibrium (GE) analysis provided by ESMs allow for the possibility that households will respond to the environmental quality changes by changing their choices in the property market. In that case, the compensating variation measure of welfare change is defined as

13 $$\begin{aligned} CV_i^{GE} =y_i^1 -e\left( {p_{j^{1}}^1 ,g_{j^{1}}^1 ,y_i^0 ,V^{0}} \right) \end{aligned}$$

In the standard property market representation when all households are renters, the quantity in (15) depends solely on a household’s baseline choice of location, $j^{0}$ and that made in the equilibrium that arises after the bypass is complete, $j^{1}$. In subsequent simulations we will examine how things change when households also select tenure in the baseline, $t^{0}$, and in the post-intervention equilibrium, $t^{1}$. The specific form for household welfare changes in the GE analysis is given by,

14 $$\begin{aligned} CV_i^{GE} =y_i^0 -\left[ {\left( {g_{j^{0}}^0 /g_{j^{1}}^1 } \right) ^{\alpha }\left( {p_{j^{1}}^1 /p_{j^{0}}^0 } \right) ^{\beta }\left( {\theta _{i,t^{0}} /\theta _{i,t^{1}} } \right) } \right] ^{\frac{1}{1-\alpha }}\left( {y_i^0 +\omega \left( {p_{j^{1}}^1 -p_{j^{0}}^0 } \right) +\pi _i^1 } \right) \nonumber \\ \end{aligned}$$

Where $\omega \left( {p_{j^{1}}^1 -p_{j^{0}}^0 } \right)$ is an endowment effect caused by property price changes for existing owners and $\pi _i^1$ represents realised capital gains following the project. The treatment of capital gains in the model is discussed in Sect. 5.2 (see Eq. 17).

Summary statistics for the welfare outcomes of the Standard Equilibrium Sorting Model (SESM) analysis are presented in the bottom panel of Table 3. Although the analysis allows for property market adjustments, a first thing to note is that the total rental revenues flowing to absentee landlords remain unchanged. That finding follows from our use of a Cobb–Douglas preference function; a function which imposes the assumption that households spend a constant proportion ($\beta _i )$ of their income on housing. While the quantity of housing units purchased by households may change as rental prices adjust, the total amount spent on housing and hence the total revenue to absentee landlords remains constant.

Things are somewhat different with regards to household welfare changes. To facilitate comparison across models, those welfare outcomes are summarised according to households’ initial residential choices; for example, the welfare outcomes listed under town centre are those experienced by households that chose to live in the town centre in the baseline even if they decided to move to the suburbs after the bypass was built. Observe that the total welfare benefits that the SESM analysis predicts will accrue to households are some five times larger than those suggested by the PE analysis; a result we might expect given that the GE analysis allows households to respond to the changed circumstances in ways which optimise their welfare. Notice also that there are significant differences in the distribution of welfare changes. According to the GE analysis renters in the town centre fare much worse than predicted by the PE analysis. At the same time, renters in the suburbs, who experienced large welfare losses under the PE analysis, are shown by the SESM analysis to realise welfare gains.

**Table 3:** Standard ESM—welfare outcomes from bypass construction

	Town centre		Suburbs
Partial equilibrium (£)			
$\Delta$ Household welfare (mean)	382.95		$-279.46$
(SD)	(926.26)		(712.76)
$\Delta$ Household welfare (total)		81,461	
$\Delta$ Rents to absentee landlords		0	
$\Delta$ Aggregate welfare		81,461	
General equilibrium (£)			
$\Delta$ Household welfare (mean)	201.66		310.63
(SD)	(498.51)		(806.51)
$\Delta$ Household welfare (total)		410,050	
$\Delta$ Rents to absentee landlords		0	
$\Delta$ Aggregate welfare		410,050	

To better understand how these differences between the analyses arise, consider Table 4 which describes the standard ESM’s prediction of the characteristics of the equilibrium established after the bypass was built.

**Table 4:** Standard ESM—neighbourhood composition after the bypass

	Town centre	Suburbs
Price	5233	5149
Population share	0.78	0.22
*Population characteristics*		
Mean Income	78,926	43,948
Mean $\beta$	0.16	0.47
*Population movements*		
From Town Renters	441	357
From Suburb Renters	802	0
Population	1243	357

As a result of reductions in traffic noise, the town centre now provides superior levels of public goods. Consequently, demand for property in the town centre increases as households from the suburbs seek to relocate to the newly improved location. Since this model assumes that supply is perfectly inelastic, property prices in the town centre are driven up while those in the suburbs fall. Despite high town-centre prices, relatively wealthy households who had previously rented in the suburbs now find their optimal choice is to rent in the town centre. In the SESM analysis, this group avoid welfare losses by moving out of the area with reduced environmental quality. Residents of the town centre fare much worse in the SESM analysis. Given their relatively low income levels, increases in town-centre rental prices are enough to drive many of this group out of that environmentally improved neighbourhood and into the environmentally deteriorated suburbs.

Accordingly, while the PE analysis predicted that the lower income households in the town centre would be advantaged by the environmental improvements in that neighbourhood, the SESM analysis paints a somewhat different picture. In particular, we find that many lower income households are displaced from the town centre by wealthier households moving in from the suburbs; as shown in Table 4 the average income of town-centre dwellers increases while that of suburban dwellers falls. As has been noted in previous applications studying air quality improvements in the LA basin (Sieg et al. 2004), Toxic Release Inventory emissions (Banzhaf and Walsh 2004) and the provision of open space (Walsh 2003), the standard ESM presentation of the property market tells a very clear story of environmental gentrification; residents of an environmentally improved area fail to gain the full benefits of those improvements as a result of rental price increases that force them out of the area to be replaced by relatively wealthy households from elsewhere. In our model while the PE analysis predicts that those in the bottom quartile of the income distribution gain an average welfare change equal to 0.26% of their income while the top quartile experiences an average welfare gain of 0.08% of their income, the SESM analysis predicts that both the top and bottom quartiles experience welfare gains of around 0.36% of income.

### Endogenous Tenure Choices (TESM)

One rather significant assumption of the standard ESM characterisation of property markets is that all households are renters. In very many locations that is not the case. In Polegate, for example, over three-quarters of the population own their home (see final row of Table 1). Following the work of Binner and Day (2015), therefore, we repeat the simulation exercise but using the ESM described in Sect. 3 in which households choose both location and tenure but maintaining the standard assumptions regarding absentee landlords and housing supply.

Allowing for homeownership introduces a further complication to the model; namely the fact that homeowner’s can experience capital gains or losses if they choose to relocate following a change of property prices in their neighbourhood. For an existing homeowner the model accounts for three ways in which capital gains or losses can accrue; (i) when a homeowner sells some but not all of their housing units and stays in the same neighbourhood and (ii) when a homeowner sells their housing in one neighbourhood and becomes an owner in a different neighbourhood, and (iii) when a homeowner sells their housing in one neighbourhood and becomes a renter. Accordingly, in the endogenous tenure ESM the budget constraint for household *i* is expressed as,

15 $$\begin{aligned} \begin{array}{lll} y_i +p_j^1 \left[ {h_{i,j^{0},O}^0 -h_{i,j^{0},O}^1 } \right] &{} =\left( {1+\delta _i m_i } \right) p_j ^{0}h_{i,j^{0},O}^0 +c^{1} &{}\quad \hbox {if selling as an owner} \\ y_i +\left[ {p_j ^{1}-\left( {1+\delta _i m_i } \right) p_j ^{0}} \right] h_{i,j^{0},O}^0 &{}=\left( {1+\delta _i m_i } \right) p_k ^{1}h_{i,k^{1},O}^1 +c^{1} &{} \quad \hbox {if moving as an owner} \\ y_i +\left[ {p_j ^{1}-\left( {1+\delta _i m_i } \right) p_j ^{0}} \right] h_{i,j^{0},O}^0 &{}=p_k ^{1}h_{i,k^{1},R}^1 +c^{1} &{} \quad \hbox {if moving to renting} \\ \end{array}\nonumber \\ \end{aligned}$$

Where the terms on the left are income and those on the right are expenditure, superscript 0 denotes a baseline choice and 1 denotes a choice made at the post-intervention equilibrium. In each equation the second expression on the left hand side denotes the capital gains or losses made on units of housing sold and the first expression on the right hand side represents the new expenditure on housing. Note that in this equilibrium sorting model households derived utility from their consumption of housing and other goods, there is no utility affect from an increase in the value of housing stock unless units are sold. Alternative models that link utility directly to changes in asset value could be specified (as in Bayer et al. 2016), however it is the authors’ opinion that the current specification is in line with real-world household preferences.

Table 5 presents details of the baseline when calibrated to the endogenous tenure ESM. The patterns of sorting are not dissimilar to those observed in the standard ESM baseline (Table 2); households with relatively lower incomes and higher preferences for larger properties tend to locate in the relatively cheaper town centre. In addition, however, we observe sorting across tenure options. Compared to owners, renters tend to have relatively lower incomes and, not surprisingly, households with relatively high preferences for homeownership, $\theta _i$, tend to choose ownership over renting.

**Table 5:** Endogenous tenure ESM—calibrated baseline neighbourhood composition

	Town centre		Suburbs	
Price	5183		5258	
Population share	0.5		0.5	
Homeownership rate	0.77		0.79	

	Renters	Owners	Renters	Owners
*Population characteristics*				
Mean income	26,610	63,233	84,672	87,953
Mean $\beta$	0.40	0.35	0.12	0.10
Mean $\theta$	1.02	1.19	1.00	1.18
Population	184	610	172	634

Importantly, the introduction of tenure into the model results in significant differences in the pattern of welfare changes experienced by households following the construction of the bypass. Those welfare outcomes are reported in Table 6. As previously, the data is organised by a household’s initial choice of location and tenure. The most notable pattern in the data is the sharp distinction in outcomes as a consequence of initial tenure status. While both homeowners and renters originally located in the town centre are, on average, advantaged by the construction of the bypass it is clear that the gains for homeowners are substantially larger than those for renters. For those originally located in the suburbs the contrast is even more distinct. While renters gain significantly from the environmental changes, those that originally owned property in the suburbs experience welfare losses.

**Table 6:** Endogenous tenure ESM—welfare outcomes from bypass construction

	Town centre		Suburbs	
	Renters	Owners	Renters	Owners
$\Delta$ Household welfare (mean)	45.43	536.13	202.19	$-57.33$
(SD)	(97.21)	(1574.00)	(601.20)	(397.33)
$\Delta$ Household welfare (total)	333,830			
$\Delta$ Rents to absentee landlords	0			
$\Delta$ Payments to mortgage lenders	2909			
$\Delta$ Aggregate welfare	336,739			

The property market adjustments that underpin those welfare outcomes are summarised in Table 7. As with the standard ESM, property prices in the town centre are driven up as households from the suburbs seek to move out of the environmentally deteriorated suburbs to the environmentally improved town centre. Those increased prices have very different impacts on the town centre residents depending on their tenure status. For many renters the increased prices offset the benefits of the environmental improvement such that a majority choose to relocate to the now cheaper suburbs. In contrast, the increase in property prices is nothing but good news for homeowners in the town centre. Such households can remain in the town centre gaining welfare from the environmental improvements or they can sell-up and use the capital gains from the sale to buy a larger property in the suburbs. For 5% of those relatively low income homeowners the latter possibility proves optimal. Either way, homeowners in the town centre can only be advantaged by the changes they experience, a very different outcome to that suggested by the standard ESM model. Tenure status also determines the options open to the relatively wealthy households in the suburbs. For renters many now find that moving away from the environmentally deteriorated suburbs to rent in the town centre is their best option, despite the higher prices. For many suburban homeowners, a move to the town centre also looks appealing though the gains from such a move are mitigated by the capital losses they suffer in selling their homes at the reduced suburban prices. Indeed, for roughly half of the suburban homeowners continuing to reside in the environmentally deteriorated suburbs turns out to be the better option. Of course, those that stay put may take advantage of the lower prices and offset their losses by moving to a relatively larger house in the suburbs. All the same, the overall outcome for the majority of suburban residents that own their home is a loss of welfare. Again, that is a distinctly different conclusion to the story told by the standard ESM treatment in which the suburban group are those that benefit most from the changes.

**Table 7:** Endogenous tenure ESM—neighbourhood composition after the bypass

	Town centre		Suburbs	
Price	5280		5165	
Population share	0.69		0.31	
Homeownership rate	0.82		0.68	

	Renters	Owners	Renters	Owners
*Population characteristics*				
Mean Income	81,668	81,719	21,203	60,427
Mean $\beta$	0.13	0.25	0.42	0.16
Mean $\theta$	1.00	1.19	1.02	1.19
*Population movements*				
From Town Renters	25	0	159	0
From Town Owners	0	581	0	29
From Suburb Renters	172	0	0	0
From Suburb Owners	0	319	0	315
Population	197	900	159	344

Notice also from Table 7, that despite many households choosing to change location, none choose to change tenure status. Indeed, that observation follows from the assumptions of our model. Since rental and purchase prices are identical within neighbourhoods, and preferences for homeownership, $$\theta _{i,t} $$, are in no way dependent on the level of provision of the public good, $$g_j $$, a household’s preferences for owning or renting will not switch no matter what the change in level of public good provision they experience. Moreover since households spend a constant proportion of their fixed income on property and all those that rented before the intervention continue to rent afterwards, it follows that revenues to absentee landlords are not changed by the property market adjustments.

**Fig. 1:**
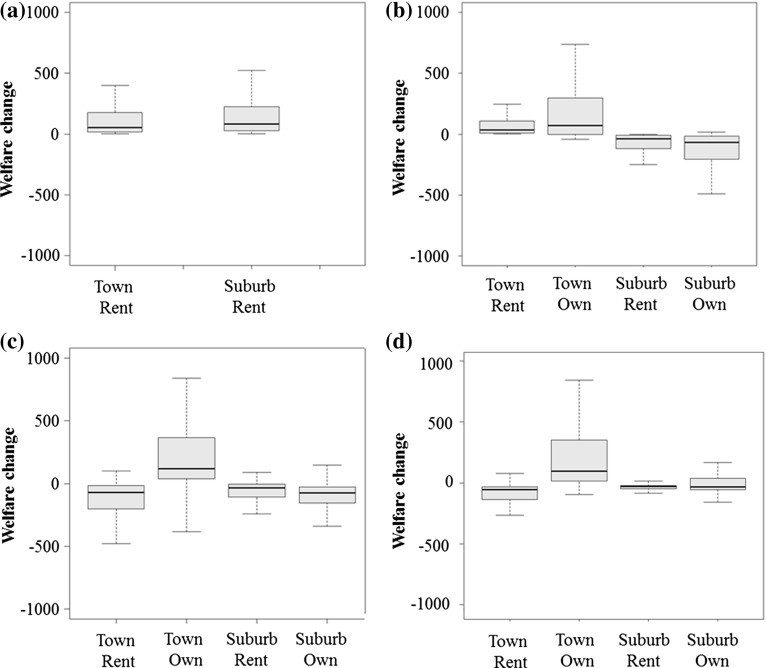
Box plots of welfare changes (£) for households grouped by initial residential choice. **a** Standard ESM, all renters. **b** ESM with tenure. **c** ESM with tenure, moving costs and housing supply realism. **d** ESM with tenure, moving costs, housing supply realism and compensation

Figure 1a and b plot out the outcomes predicted by the standard ESM and endogenous tenure ESM respectively. The figures group households by their initial residential choices and present boxplots of the welfare changes each group experiences. The solid line within each box represents the median value whilst the top and bottom of the box are the $50{\mathrm{th}}$ and $25{\mathrm{th}}$ percentiles respectively; similarly the top and bottom of the dashed line are the $95{\mathrm{th}}$ and $5{\mathrm{th}}$ percentiles. The standard ESM anticipates welfare gains across both town centre and suburban residents with the relatively larger gains being amassed by the wealthier suburban residents through a process of environmental gentrification. The endogenous tenure ESM illustrates the misleading nature of that narrative. Tenure status is fundamental to welfare outcomes in a property market. While renters in the town centre are indeed disadvantaged by the process of environmental gentrification, homeowners are not. They directly benefit from the rising prices that drive renters out through the increased value of their home. In contrast, for relatively wealthy suburban homeowners a combination of a deteriorated environment and the falling value of their home precipitates substantial welfare losses.^8^

### Housing Supply Realism and Moving Costs (TESM+)

In the simulations discussed thus far we have assumed that there are no moving costs and followed the standard ESM assumptions regarding housing supply, these are two important sources of friction that play out differently through the property market (see Appendix B for further discussion of the distinction between moving costs and repackaging costs). Moving costs have been explored in previous studies, however the impacts of repackaging costs and capacity constraints have not. Consequently, in this section we adopt a fixed moving cost and focus on the impact of housing supply constraints. The fixed moving cost is set at 200, approximately 5% of per unit property prices.^9^

**Table 8:** Initial and post-intervention property sizes

Model	Neighbourhood	Mean housing units per property (SD)	
		Baseline	After Bypass
*Standard ESM*			
	Town centre	4.17 (9.67)	2.67 (7.43)
	Suburb	1.93 (6.09)	4.35 (10.19)
*Tenure ESM*			
	Town centre	4.01 (9.36)	2.91 (6.70)
	Suburb	2.06 (6.58)	3.30 (10.66)
*Tenure ESM—supply side realism and moving costs*			
	Town centre	4.01 (9.35)	3.47 (7.24)
	Suburb	2.06 (6.58)	2.39 (9.04)

While the standard approach to modelling housing supply is mathematically convenient and can approximate certain forms of adjustment including such as depreciation and renovation of properties, it does not adequately reflect the realities of accommodating changing populations within a neighbourhood. In this regard, the standard approach assumes that we can move costlessly for example from a neighbourhood of *N* households each with a property offering *X* / *N* units of the housing index to a neighbourhood of $N+1$ households each enjoying a property offering $X/\left( {N+1} \right)$ units of the housing index. In other words, new households can be accommodated within the current housing stock without cost. We refer to this as costless repackaging. One way we might justify the costless repackaging assumption is by imagining that all increases in the population are accommodated within a neighbourhood by house sharing. Of course, the empirical reality is one in which we also observe significant costly investment in the development of new single-household occupancy properties; be that through the division and remodelling of the existing stock, through demolition and rebuilding or through infilling. In this section we develop a housing supply function is designed to better reflect that reality; that there is a real cost involved in creating an extra property in a neighbourhood and providing that property with the principal facilities and essential service connections that are required for occupation by a household.

One possible short-coming of standard ESM housing supply assumptions can be seen in the changes in populations predicted by those models. Compare, for example, the baseline population in the town centre of 798 households (Table 2) with that predicted by the standard ESM at the equilibrium after the bypass of 1243 households (Table 4). The model allows for an almost 60% increase in town centre inhabitants. Not dissimilar outcomes arise in the endogenous tenure ESM’s described in the last two sections. In reality, the number of households that can be accommodated in a spatially-defined neighbourhood is limited by the number of new properties that can be constructed within that limited space or reconfigured from the existing property stock. The manner in which the standard ESM assumptions allow for this expanded population is illustrated in the top rows of Table 8. Observe that in the baseline, the fixed stock of housing units in the town centre is configured into properties that on average contain 4.17 housing units. After the bypass, that average drops to 2.67 housing units. In other words, a larger population is accommodated by repackaging housing units into new properties of increasingly modest size (in terms of homogenous quality units). Again, the assumptions that lead to that outcome are questionable. First, one would expect that the higher prices that accompany increased demand in the town centre would encourage some expansion of the housing stock perhaps through infilling on previously undeveloped land. Second, one might expect that the process whereby housing units are reconfigured to generate new smaller properties would be associated with some cost; the more properties one wished to configure out of a fixed number of housing units, the higher the cost of their supply.

To explore the implications of the standard ESM’s supply-side assumptions, we further augment our ESM by specifying a housing supply function that adheres to the following plausible assumptions; the supply function (i) is elastic such that more housing units can be constructed but with increasing marginal costs, (ii) has a capacity constraint for the total quantity of properties that can be accommodated within the spatially-constrained geographic extent of a neighbourhood and (iii) includes a cost associated with repackaging housing units to create properties of a different size.

16 $$\begin{aligned} H_j ^{s}=H_j^0 +a\frac{\left( {p_j -p_j^0 } \right) }{p_j^0 }H_j^0 -b\frac{\left| {n_j -n_j^0 } \right| }{n_j^0 }H_j^0 \quad \left( {0\le n_j \le D_j n_j^0 } \right) \end{aligned}$$

where $$n_j $$ and $$n_j^0 $$ are the new and initial population size of neighbourhood *j* respectively, $$H_j^0 $$ is the baseline housing supply, *a* and *b* are constants relating to the price elasticity of housing supply and the marginal cost of using housing units to construct a new property. The price elasticity of housing was set to a value of 0.5 in accordance with empirical estimates (Swank et al. 2002; Meen 2005). In the absence of empirical data upon which to define the costs of repackaging housing units, we explored a range of values for$$\hbox { }b$$, the results were robust for values ranging from 0.2 to 2, in the simulations presented below we set *b* to 0.5, implying that a 1% change in the population imposes repackaging costs that reduce available housing units by 0.5%.

Observe that housing supply is defined within the limits $$ 0\le n_j \le D_j n_j^0 $$, where $$D_j $$ represents the maximum percentage increase in the number of properties (and hence the population) that can be supported by neighbourhood *j*. For the purposes of our simulations, $$D_j $$ is set as a uniform capacity limit that constrains the development of new properties to a maximum of fifteen per cent in each neighbourhood. The introduction of a fixed capacity limit in the supply of housing units alters the nature of the housing market. When there is no capacity limit property prices are able to adjust until the point at which demand is equal to supply. However, with a capacity limit, reducing the price may lead to an increase in demand but also to the total number of people demanding property in the area may exceed the capacity limit.^10^

While changing the housing supply function has no impact on the baseline (Table 5), as shown in Table 9, the equilibrium that establishes after the bypass is constructed is somewhat different. Observe that in this simulation the town centre capacity constraint, which in this case limits that neighbourhood’s population share to 0.57, is binding. As a result prices in the town centre are pushed higher than in previous models as wealthier households compete for access to the limited number of properties that can be accommodate in that neighbourhood. As can be seen in the population movements section of Table 9, the inflation of town centre prices leads this model to predict a greater displacement of households from the centre to the suburbs than in models in which capacity limits are not accounted for. Consequently, there is an increase in demand for housing units in the suburbs, which tends to support property prices in that neighbourhood despite the small fall in public good provision.

**Table 9:** Endogenous tenure ESM with housing supply realism—neighbourhood composition after the bypass

	Town centre		Suburbs	
Price	5375		5258	
Population Share	0.57		0.43	
Homeownership Rate	0.79		0.76	

	Renters	Owners	Renters	Owners
*Population characteristics*				
Mean Income	81,321	92,427	23,661	52,977
Mean $\beta$	0.34	0.30	0.16	0.13
Mean $\theta$	1.00	1.18	1.01	1.19
*Population movements*				
From Town Renters	159	0	25	0
From Town Owners	0	586	1	23
From Suburb Renters	32	0	140	0
From Suburb Owners	0	136	0	498
Population	191	722	166	521

These price effects have important and substantial implications for welfare changes, as summarised in Table 10. The relatively higher prices in both neighbourhoods predicted by this model result in an average welfare loss for renters initially located in both neighbourhoods. In contrast, those higher prices confer gains to owners in the town centre and provide a source of compensation for owners in the suburbs who in previous models had endured capital losses. Consequently, the distribution of welfare gains and losses is transformed once the housing supply adjustments are incorporated; with large losses accruing to lower income renters and large gains to wealthy owners. Figure 1c plots the distribution of welfare changes for each of the initial residential choice groups. Comparing Fig. 1c to b, it is apparent that the introduction of a more realistic housing supply response leads to predictions of property market adjustments that tend to further redistribute the welfare gains from the environmental changes towards homeowners and away from renters. While the particular pattern of welfare changes suggested by this model are, of course, dependent on the particular conditions described by this simulation, the clear lesson is that housing supply responses to localised environmental change are fundamental to the distribution of welfare outcomes resulting from that change.

### Compensation

In the UK, following public works that lead to a reduction in the value of properties, the owners of those properties (but not the renters) are entitled to claim compensation under Part 1 of the Land Compensation Act 1973. According to that policy owners of properties in the suburbs that are exposed to greater noise pollution as a result of the bypass are legally entitled to compensation equal to the loss in market value of their property. Importantly, that compensation is paid according to the fall in price that would be expected for a property in the absence of relocation and other property market adjustments, rather than the change in price actually realised. Our intuition is that compensation policies of this type have the distortionary effect of protecting homeowners against potential capital losses while pushing the burden of environmental degradation onto the rental sector. As a final exercise, we use our enhanced ESM to consider the implications of a policy intervention relevant to our case study location.

Following the procedure used to calculated Part 1 compensation claims, we incorporate a compensation mechanism defined as follows,

17 $$\begin{aligned} compensation_{i,j,O} =\left\{ {{\begin{array}{ll} 0&{} \quad \hbox {if} \ \frac{\partial p^{0}}{\partial g_2 }*\Delta g_{j,2} *h_{i,j}^0 <50 \\ {\frac{\partial p^{0}}{\partial g_2 }*\Delta g_{j,2} *h_{i,j,O}^0 }&{} \quad \hbox {if} \ \frac{\partial p^{0}}{\partial g_2 }*\Delta g_{j,2} *h_{i,j}^0 \ge 50 \\ \end{array} }} \right. \end{aligned}$$

The level of compensation is calculated from the implicit price of environmental quality in the economy in the baseline, $$\partial p^{0}/\partial g_2 $$. Multiplying the implicit price by the size of the change in environmental quality, $$\Delta g_{j,2} $$, and then again by the quantity of housing in a homeowner’s property, $$h_{i,j,O}^0 $$, provides a figure likely to resemble that which might be calculated by an assessor attempting to estimate the anticipated change in value of a property as a result of the public works. Following the 1973 Compensation Act the minimum level of value change eligible for compensation is £50.

**Table 10:** Endogenous tenure ESM with housing supply realism—welfare outcomes

	Town centre		Suburbs	
	Renters	Owners	Renters	Owners
$\Delta$ Household welfare (mean)	$-187.76$	522.91	$-78.47$	$-124.95$
(SD)	(305.8)	(1568.0)	(466.2)	(404.7)
$\Delta$ Household welfare (total)	191,710			
$\Delta$ Payments to mortgage lenders	6822			
Moving costs	43,400			
$\Delta$ Aggregate welfare	198,532			

Again, the relevant baseline is given in Table 5. Table 11 presents the prices and population characteristics following the construction of the bypass. The largest compensation payment is £6980. The receipt of compensation has little impact on the residential choices of households; as with rental revenues, households tend to use the compensation to increase the number of housing units and other goods that they consume. The influence of compensation is most clearly visible in Table 12 and Fig. 1d, which summarise the welfare changes by groups. Comparing with Table 10, on average, the payments more than compensate homeowners initially located in the suburbs whose properties are negatively impacted by the new bypass. As they do not receive compensation, most renters continue to experience a reduction in welfare. Comparing Fig. 1d to c, compensation clearly alters the distribution of gains and losses, broadening the division between renters and owners. In both neighbourhoods, the welfare changes experienced by the majority of renters are negative while those for owners are almost entirely positive. While the standard ESM predicted the lowest quartile of the income distribution would gain welfare gains equivalent to a 0.26% rise in income resulting from the change, once the role of tenure and compensation have been incorporated in the analysis, these gains disappear giving way to average welfare losses equal to 0.5% of income.

**Table 11:** Endogenous tenure ESM with housing supply realism, moving costs and compensation—neighbourhood composition after the bypass

	Town centre		Suburbs	
Price	5376		5258	
Population Share	0.57		0.43	
Homeownership Rate	0.79		0.76	

	Renters	Owners	Renters	Owners
*Population characteristics*				
Mean Income	81,322	92,428	23,661	52,977
Mean $\beta$	0.34	0.30	0.16	0.13
Mean $\theta$	1.00	1.18	1.01	1.19
*Population movements*				
From Town Renters	159	0	25	0
From Town Owners	0	586	1	23
From Suburb Renters	32	0	140	0
From Suburb Owners	0	136	0	498
Population	191	722	166	521

**Table 12:** Endogenous tenure ESM with housing supply realism, moving costs and compensation—welfare outcomes

	Town centre		Suburbs	
	Renters	Owners	Renters	Owners
$\Delta$ Household welfare (mean)	$-187.76$	522.92	$-78.47$	73.14
(SD)	(305.8)	(1568. 0)	(466.2)	(513.0)
$\Delta$ Household welfare (total)	317,300			
$\Delta$ Payments to mortgage lenders	6851			
Moving costs	43,400			
Compensation	(131,620)			
$\Delta$ Aggregate welfare (net of compensation)	367,300			

## Concluding Remarks

The central objective of this paper has been to explore the insights that ESM models can provide in predicting the welfare implications of projects that result in localised changes in environmental quality. Currently, standard practice in the appraisal of such projects is to establish the expected welfare changes experienced by those directly impacted by the project using methods of non-market valuation that are nearly exclusively static in nature. While correct in the short run, such PE analyses do not allow for the fact that households may respond to an exogenous policy change through altering their residential choices in the property market. ESMs provide one way of exploring those GE responses.

Our research suggests that policy appraisals based on PE analyses, ignoring the property market’s role in reallocating access to public goods, are likely to significantly underestimate the aggregate welfare impacts of a project and provide highly misleading information regarding its distributional impacts. In our simulations, the PE analysis suggests that the project most greatly benefits the poorer households located in the improved town centre, a fact that means that the lowest income quartile achieve welfare gains equivalent to a 0.26% rise in income in comparison to a 0.08% rise for the highest income quartile. Once we account for adjustments in the property market, even in a simple ESM ignoring tenure status, this result does not hold. According to the standard ESM, wealthier households are able to exploit their superior buying power in property markets to recover their welfare losses by moving into improved neighbourhoods. The demand of these in-comers tends to increase property prices in improved areas which, in turn, disadvantages the poorer residents of those neighbourhoods. Ultimately, the standard ESM suggests that, in terms of shares of total utility, the policy results in a situation that is no more equitable than in the baseline, in fact one which is mildly regressive. Indeed the predictions of that model is that, on average, the lowest income quartile experience welfare losses equivalent to a 0.5% reduction in income, whilst the top income quartile benefit from welfare gains averaging 0.3% of their incomes.

A central contribution of the research in this paper has been to examine whether the narrative of environmental gentrification suggested by the standard ESM is maintained when greater realism is introduced into the model’s representation of the property market, for example, in allowing households to choose whether to rent or own their property. The endogenous tenure ESM we explore in this paper confirms that ignoring tenure choice is a very significant omission. As anticipated, one of the key differences we observe between renters and owners is that owners are more greatly benefited by neighbourhood improvements. In particular, those improvements increase demand in the neighbourhood and force up prices. Renters lose out when prices increase as their rents rise. Homeowners, in contrast, gain from price increases through the escalating value of their property. In contrast, in neighbourhoods that endure deteriorating environmental quality the opposite pattern emerges. As demand falls, prices fall, saddling homeowners with capital losses but renters with reduced rental costs. Accordingly, only one part of the story is told by narratives of environmental gentrification. By ignoring homeownership, such conclusions fail to distinguish the crucial role of tenure in determining patterns of welfare distribution.

Interestingly as we augment our model to capture more real world complexity, we see a systematic switch away from the pattern of renters doing well when owners do badly and vice versa. For example, when we specify a housing supply function that restricts the number of properties that can be built in any one area, we find that constraint binding in the improved town centre. As a result, prices in that neighbourhood rise steeply. Those price increases are clearly bad news for renters currently resident in the town centre but they are also bad news for renters living in the suburbs since they significantly reduce the benefits of escaping the environmental deterioration in the suburbs by moving into the town centre. Moreover, when we introduce a policy (modelled on real world analogues) to compensate homeowners in the suburbs for the loss in value of their property we find a very different pattern emerges. The GE analysis predicts that current homeowners generally benefit from the project while, in the main, renters experience welfare losses.

While the emergent pattern observed in our illustrative case study is not generalizable, the key insight provided by the simulations is that the manner in which an ESM captures the mechanics of the property market has significant implications for its predictions of the distributional consequences of policies resulting in localised changes in environmental quality. In particular, our simulations demonstrate the pivotal role played by tenure choice and housing supply response in shaping welfare outcomes.

Our work suggests a number of important areas of research that demand attention if ESMs are to fulfil their potential in advising policy makers on the welfare implications of policies resulting in localised environmental change. First, ESM models must develop a richer representation of the tenure choices available to households in the property market. While our work has focused on the core dichotomy of renting and owning, another significant option is provided by tenancy in publicly-owned housing. Likewise, to truly capture the complexity of tenure choices in property markets, ESMs will have to develop more realistic representations of the financing options open to households in purchasing a property. Again our work has made some advance in that direction, but there is much room for progress. A second key area in need of further research concerns the representation of housing supply responses in ESM models. We have shown that making a limited number of relatively plausible improvements to the standard supply function used in ESM analyses has significant implications for the model’s predictions, however our specification is calibrated but not estimated using empirical data and fails to capture important realities particularly those relating to planning laws. While our results show that supply side constraints and moving costs play a pivotal role in shaping the distribution of welfare gains and losses, if we want to fully utilize these types of analyses in advising policy makers, an investment in collecting and maintaining rich datasets for model estimation in crucial.

Despite the limitations of standard ESM modelling assumptions highlighted in this paper, a simple truth remains: current reliance of policy appraisal on PE methods is fundamentally problematic and can result in highly misleading predictions of the welfare implications of local environmental change. ESMs provide perhaps the only tool through which those problems might eventually be overcome.

## Electronic supplementary material

Below is the link to the electronic supplementary material.

Supplementary material 1 (docx 17 KB)

## Footnotes

1 It is possible to extend the model to allow preferences for homeownership to evolve over time, for example as households mature, and are influenced by experiences including previously ownership and house prices observations. In our case study application we assume that each household’s preferences for homeownership are fixed.

2 In this paper we assume that the mortgage interest rate and the loan to value ratio are exogenous, however it is possible to extend the model to endogenise them, for example by allowing households to use capital gains to lower their loan to value ratio. We anticipate that doing so would serve to increase the welfare disparity between renters and owners.

3 In undertaking this research we experimented with other models in which we treated purchase and rental sectors as independent markets with separate supply functions, an outcome that might arise if government regulations prevented the transfer of properties between sectors. While we found a high degree of price convergence between rent and purchase prices, not surprisingly we also found that assuming a different structure to the property market altered predictions of the model. In reality, purchase and rental markets do not exist separately, though the closeness of their interdependence is mediated by government regulations that may restrict allowable tenure types and by differences in taxation and transaction costs encountered in the two sectors. Ultimately, real property markets exist as some compromise between the two extremes of pure arbitrage and independent markets. While our work suggests that the predictions of an ESM will be shaped by assumptions regarding the interdependence of purchase and rental markets, we do not pursue that avenue of research further in this paper. Rather, as will be evident subsequently, our focus is on the relaxation of the standard ESM assumption that housing units can be costlessly repackaged and how allowing for more sophisticated housing supply responses changes predictions of the distributional consequences of localised environmental change.

4 This is not a restriction of ESMs in general which can encompass a variety of government interventions; for example levying taxes, providing subsidies, funding expenditure on public goods, or setting production standards.

5 The Matlab code is available from the authors upon request.

6 The simulations were run multiple times using both pre-bypass prices and randomly selected initial price vectors for the algorithm in order to check for the existence of multiple equilibria. In each simulation the results converge to a single equilibrium. Although this does not prove uniqueness of the equilibrium it is an encouraging result.

7 For interested readers, the partial equilibrium equation for compensating variation has been provided in Appendix A in the online supplementary information.

8 Appendix B in the online supplementary information presents results from the TESM with the inclusion of fixed moving costs. The introduction of these moving costs creates friction in the market and reduces the amount of relocation, which has a moderate impact on property prices. However, the results show that despite this friction tenure status remains fundamental to welfare outcomes.

9 A range of uniform and asymmetric moving costs were explored. While the welfare of individual households is affected by these moving costs, these impacts are counteracted by the impact of property prices such that the net impact on the distribution of welfare across renters and owners, in the town and suburbs is not (statistically) significantly impacted, even for large symmetric moving costs (e.g. 20% of property price per unit).

10 To solve the model when the capacity limit is binding it is necessary to determine how housing suppliers decide which prospective buyers to sell to. We assume that subject to the market price per unit of housing in the area suppliers of housing choose to sell (or rent) to households who are willing to purchase the largest quantity of housing units from them until the population capacity limit has been reached.  This process ensures that the maximum number of housing units (up to the total quantity supplied in the market) are sold (or rented) subject to the capacity limit.

## References

[CR1] Banzhaf HS, Walsh RP (2004) Testing for environmental gentrification: migratory responses to changes in environmental quality. AERE Workshop, Estes Park, CO

[CR2] Bayer P, Mcmillan R, Rueben K (2004) An equilibrium model of sorting in an urban housing market. NBER Working Paper

[CR3] Bayer P, Keohane N, Timmins C (2009). Migration and hedonic valuation: the case of air quality. J Environ Econ Manag.

[CR4] Bayer P, Mcmillan R, Murphy A, Timmins C (2016). A dynamic model of demand for houses and neighborhoods. Econometrica.

[CR5] Binner A (2012) Spatial processes in environmental economics: empirics and theory. Doctoral thesis, University of East Anglia

[CR6] Binner A, Day B (2015) Exploring mortgage interest deduction reforms: an equilibrium sorting model with endogenous tenure choice. J Public Econ 122(C):40–54

[CR7] Carbone J, Smith V (2008). Evaluating policy interventions with general equilibrium externalities. J Public Econ.

[CR8] Day B, Bateman I, Lake I (2007). Beyond implicit prices: recovering theoretically consistent and transferable values for noise avoidance from a hedonic property price model. Environ Resour Econ.

[CR9] Dietz R (2003) The social consequences of homeownership. Homeownership Alliance, Report 16

[CR10] Dietz R, Haurin D (2003). The social and private micro-level consequences of homeownership. J Urban Econ.

[CR11] Ellickson B (1971). Jurisdictional fragmentation and residential choice. Am Econ Rev.

[CR12] Epple D, Ferreyra MM (2008). School finance reform: assessing general equilibrium effects. J Public Econ.

[CR13] Epple D, Platt G (1998). Equilibrium and local redistribution in an urban economy when households differ in both preferences and incomes. J Urban Econ.

[CR14] Epple D, Romer T (1991). Mobility and redistribution. J Political Econ.

[CR15] Epple D, Romer T, Sieg H (2001). Interjurisdictional sorting and majority rule: an empirical analysis. Econometrica.

[CR16] Epple D, Romano R, Sieg H (2010) The intergenerational conflict over the provision of public education. Working Paper

[CR17] Ferreira F (2010). You can take it with you: proposition 13 tax benefits, residential mobility, and willingness to pay for housing amenities. J Public Econ.

[CR18] Ferreyra MM (2007). Estimating the effects of private school vouchers in multidistrict economies. Am Econ Rev.

[CR19] Fernandez R, Rogerson R (1998). Public education and income distribution: a dynamic quantitative evaluation of education-finance reform. Am Econ Rev.

[CR20] Financial Services Authority (2007) Mortgage Product Sales Data Trends Report

[CR21] Goulder LH, Roberton C, Williams I (2003). The substantial bias from ignoring general equilibrium effects in estimating excess burden, and a practical solution. J Political Econ.

[CR22] Green R, White M (1997). Measuring the benefits of homeowning: effects on children. J Urban Econ.

[CR23] Hallstrom DG, Smith VK (2003) Habitat protection policies and open space: a general equilibrium analysis of “Takings” and “Givings”. Preliminary Draft. North Carolina State University

[CR24] Haurin DR, Parcel TL, Haurin RJ (2002). Does homeownership affect child outcomes?. Real Estate Econ.

[CR25] Highways Agency (1991) A27 Polegate Bypass Environmental Statement. Area 4: Archaeological Reports. Highways Agency

[CR26] Highways Agency (2009) Post opening project evaluation five year after study: A27 Bypass. Department for Transport

[CR27] Kerry Smith V, Sieg H, Spencer Banzhaf H, Walsh RP (2004). General equilibrium benefits for environmental improvements: projected ozone reductions under EPA’s prospective analysis for the Los Angeles air basin. J Environ Econ Manag.

[CR28] Klaiber AH, Phaneuf DJ (2010). Valuing open space in a residential sorting model of the Twin Cities. J Environ Econ Manag.

[CR29] Kuminoff NV (2009). Decomposing the structural identification of non-market values. J Environ Econ Manag.

[CR30] Kuminoff NV (2011) An intraregional model of housing and labor markets for estimating the general equilibrium benefits of large changes in public goods. Mimeo

[CR31] Kuminoff N, Smith V, Timmins C (2010) The new economics of equilibrium sorting and its transformational role for policy evaluation. NBER Working Paper

[CR32] Lagarias JC, Reeds JA, Wright MH (1998). Convergence properties of the Nelder—Mead simplex method in low dimensions. SIAM J Optim.

[CR33] Liu F (2000). Environmental justice analysis: theories, methods, and practice.

[CR34] Meen G (2005). On the economics of the Barker review of housing supply. Hous Stud.

[CR35] Mills ES (1967). An aggregative model of resource allocation in a metropolitan area. Am Econ Rev.

[CR36] Nechyba T (2003) Introducing school choice into multidistrict public school systems. National Bureau of Economic Research, The Economics of School Choice

[CR37] Oates WE (1969). The effects of property taxes and local public spending on property values: an empirical study of tax capitalization and the tiebout hypothesis. J Political Econ.

[CR38] Poustie M (2004) Environmental justice in SEPA’s environmental protection activities: a report for the Scottish Environment Protection Agency. University of Strathclyde Law School

[CR39] Rohe WM, Leslie SS (1996). Homeownership and neighborhood stability. Hous Policy Debate.

[CR40] Rohe WM, Zandt SV, Mccarthy G (2001) The social benefits and costs of homeownership: a critical assessment of the research. Low-Income Homeownership Working Paper Series, pp 1–35

[CR41] Schelling TC (1969). Models of segregation. Am Econ Rev.

[CR42] Sieg H, Smith V, Banzhaf H, Walsh R (2002). Interjurisdictional housing prices in locational equilibrium. J Urban Econ.

[CR43] Sieg H, Smith KV, Banzahf HS, Walsh RP (2004). Estimating the general equilibrium benefits of large changes in spatially delineated public goods. Int Econ Rev.

[CR44] Smith VK, Carbone JC (2007). Should benefit-cost analyses take account of general equilibrium effects?. Res Law Econ.

[CR45] Smith VK, Sieg H, Spencer Banzhaf H, Walsh R (2004). General equilibrium benefits for environmental improvements: projected ozone reductions under EPA&apos;s prospective analysis for the Los Angeles air basin. J Environ Econ Manag.

[CR46] Swank J, Kakes J, Tieman A (2002) The housing ladder, taxation and borrowing constraints, Research Memorandum 688. Netherlands Central Bank, Research Department

[CR47] Tiebout CM (1956). A pure theory of local expenditures. J Political Econ.

[CR48] Tra CI (2010). A discrete choice equilibrium approach to valuing large environmental changes. J Public Econ.

[CR49] Walker G (1998). Environmental justice and the politics of risk. Town Ctry Plan.

[CR50] Walker G, Bickerstaff K, (2000) Polluting the poor: an emerging environmental justice agenda for the UK? Critical Urban Studies occasional papers, Centre for Urban and Community Research, Goldsmiths College, University of London, London

[CR51] Walsh R (2003). Analyzing open space policies in a locational equilibrium model with endogenous landscape amenities.

[CR52] Walsh R (2007). Endogenous open space amenities in a locational equilibrium. J Urban Econ.

[CR53] Walton W, Shaw J (2003). Applying the new appraisal approach to transport policy at the local level in the UK. J Transp Geogr.

