# Supplementary material for: How Property Markets Determine Welfare Outcomes: An Equilibrium Sorting Model Analysis of Local Environmental Interventions
Source: Environ Resour Econ (Dordr). 2017 Feb 13;69(4):733–61. doi: 10.1007/s10640-016-0101-8 (PMC6560932; doi:10.1007/s10640-016-0101-8)
Supplement: Supplementary file 1 — Supplementary material 1 (docx 17 KB) [file 10640_2016_101_MOESM_ESM.docx]

**Appendix A: Partial and General Equilibrium Welfare Measures**

The PE measure of welfare change for household$i$, initially resident in neighbourhood $j^{0}$, can be defined by the compensating variation measure,

| ${CV}_{i}^{PE}=y_{i}^{0}-e(p_{j^{0}}^{0},g_{j^{0}}^{1}\boldsymbol{,}y_{i}^{0},V^{0})$ |  |
| --- | --- |

where $e(\cdot)$ is the expenditure function, the superscript 0 identifies variables prior to the bypass, superscript 1 to variables after construction and $V^{0}$is the utility level realised from the optimal residential bundle in the baseline (see equation 8). Replacing using our particular functional form assumptions gives;

| ${CV}_{i}^{PE}=\left[ 1-\left( {g_{j^{0}}^{0}}/{g_{j^{0}}^{1}} \right)^{\frac{\alpha}{1-\alpha}} \right](y_{i}^{0}-p_{j_{0}}^{0}\omega_{0})$ |  |
| --- | --- |

**Appendix B: Moving Costs**

As was discussed in section 5.3 of the paper, supply side constraints are fundamentally different from moving costs in both in principle and mathematically. These costs play out differently through the property market. Moving costs affect demand and apply to households who relocate, the magnitude of moving costs may vary across households and differ depending on whether a household is relocating in the same area or not, or whether they alter their tenure status. Mathematically moving costs enter the household budget constraint and can be avoided by choosing not to relocate. In contrast, supply side constraints affect the supply of properties in the market and affect everyone locating in the affected area. In our paper, the supply side constraints apply as a result of repackaging and physical limits.

To illustrate this Table A.1 presents results from a tenure ESM with fixed moving costs of £200 which are incurred by a household who moves from one neighbourhood to another or changes tenure but no housing supply constraints. The results serve to highlight the way in which the housing supply constraints introduced in our paper shape the patterns of adjustment and new equilibrium following the construction of the bypass. In the absence of supply side constraints a larger share of the population locates in the town following the construction of the bypass. This occurs because repackaging costs are not taken into account and homogenous quality units of housing are redistributed amongst a larger number of households. Consequently, lower income households can reduce their housing consumption and remain in the town, offering existing units of housing to higher income households who relocate in the town from the suburbs. The absence of repackaging costs and a capacity constraint leads to a smaller price rise in the town (relative to Table 9) and, as a result, a lower rate of relocation from the town to the suburbs such that the price there is predicted to fall. This prediction is not aligned with the empirical observations for housing prices in Polegate. Moreover, the model predicts a slightly larger increase in population share in the town centre than is observed in the census data. As a result, the model with only moving costs fails to capture the negative welfare impacts on renters associated with larger price rises in the town and price rises in the suburbs.

**Table 13: Endogenous tenure ESM with fixed moving costs and compensation**

**- Neighbourhood composition after the bypass**

|  | **Town Centre** | | **Suburbs** | |
| --- | --- | --- | --- | --- |
| Price | 5285 | | 5,169 | |
| Population Share | 0.58 | | 0.42 | |
| Homeownership Rate | 0.78 | | 0.77 | |
|  | **Renters** | **Owners** | **Renters** | **Owners** |
| *Population Characteristics:* | | | | |
| Mean Income | 76,271 | 92,367 | 25,992 | 52,732 |
| Mean $\beta$ | 0.34 | 0.30 | 0.16 | 0.13 |
| Mean $\theta$ | 1.01 | 1.18 | 1.00 | 1.19 |
| *Population Movements:* | | | | |
| From Town Renters | 169 | 0 | 15 | 0 |
| From Town Owners | 0 | 587 | 0 | 23 |
| From Suburb Renters | 34 | 0 | 138 | 0 |
| From Suburb Owners | 0 | 137 | 0 | 497 |
| Population | 203 | 724 | 153 | 520 |

**Table 14: Endogenous tenure ESM with fixed moving costs and compensation**

**– Welfare outcomes**

|  | **Town Centre** | | **Suburbs** | |
| --- | --- | --- | --- | --- |
|  | **Renters** | **Owners** | **Renters** | **Owners** |
| $\Delta$ Household welfare (mean) | 18.11 | 518.08 | 141.18 | -123.33 |
| (Std. Dev.) | (107.6) | (1,535.5) | (620.1) | (400.4) |
| $\Delta$ Household welfare (total) | 265,450 | | | |
| $\Delta$ Payments to mortgage lenders | 2,059 | | | |
| Moving costs | 41,800 | | | |
| $\Delta$ Aggregate welfare | 267,509 | | | |
